# Supplementary material for: Spreading ridge migration enabled by plume-ridge de-anchoring
Source: Nat Commun. 2024 Oct 16;15:8934. doi: 10.1038/s41467-024-53397-w (PMC11484986; doi:10.1038/s41467-024-53397-w)
Supplement: Supplementary file 1 — Supplementary Information [file 41467_2024_53397_MOESM1_ESM.pdf]

Spreading ridge migration enabled by plume-ridge de-anchoring

Ben Mather, Maria Seton, Simon Williams, Joanne Whittaker, Rebecca Carey,
Maëlis Arnould, Nicolas Coltice and Robert Duncan

SUPPLEMENTARY INFORMATION

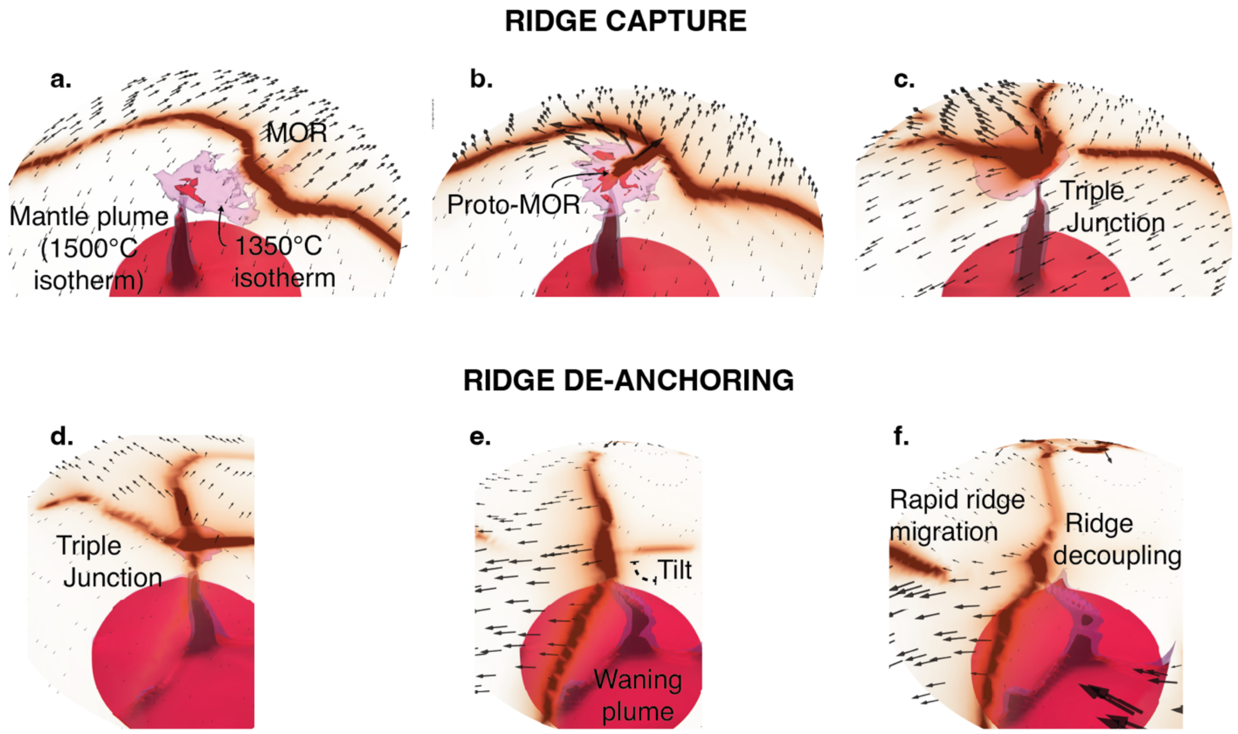

Supplementary Fig. 1: Model snapshots detailing the key stages of ridge capture and ridge de-anchoring. Model snapshots of plume-ridge interactions extracted from 3D numerical models of whole-mantle convection correspond to the illustrations presented in Figure 2. They depict three stages of ridge capture (a–c) and de-anchoring (d–f) for two separate plume-ridge interactions. Each timestep is equivalent to approximately 1 Myr and is equivalent to the timeseries presented in Figure 3. **a.** Timestep 20 of 100; **b.** Timestep 30 of 100; **c.** Timestep 40 of 100 **d.** Timestep 40 of 100; **e.** Timestep 75 of 100; **f.** Timestep 90 of 100. The full timeseries can be viewed as a video in Supplementary Movie 1 for a–c and Supplementary Movie 2 for d–f.

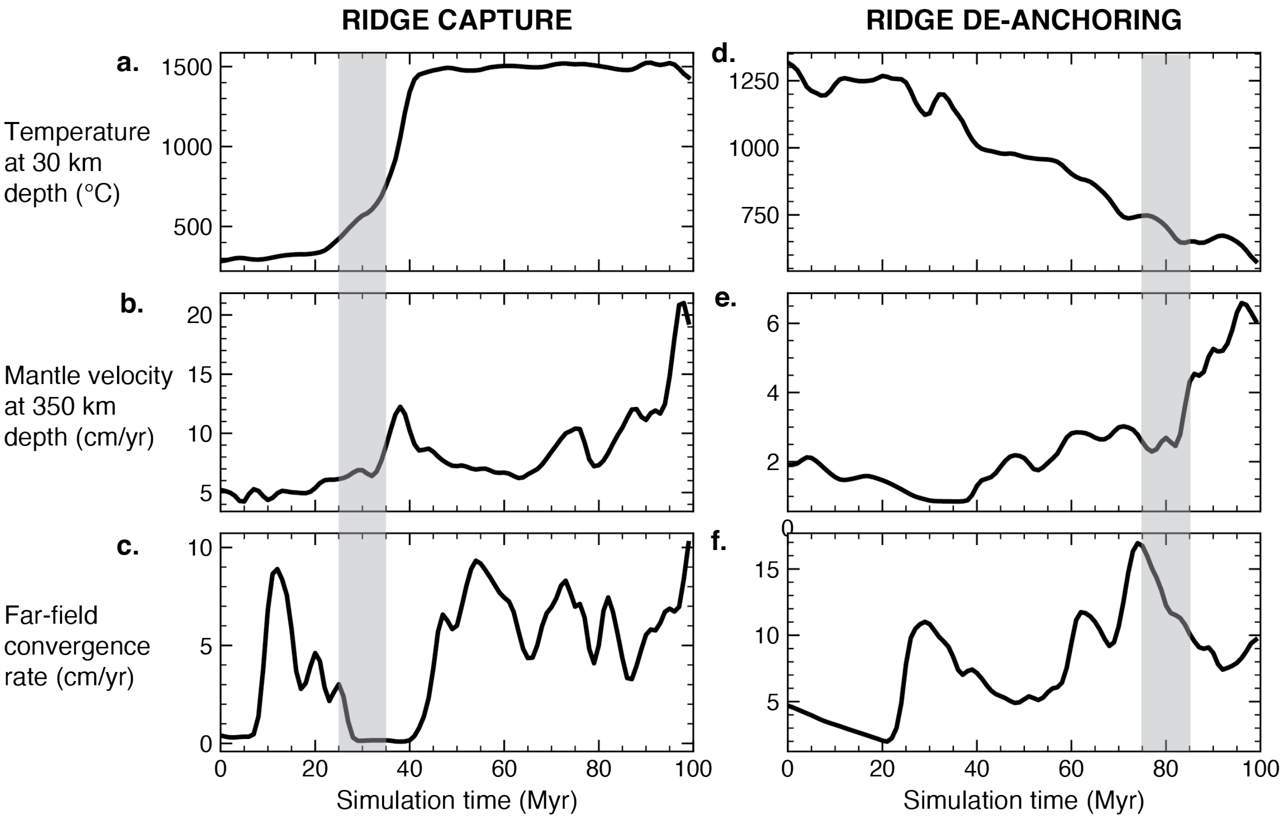

Supplementary Fig. 2: Extended ridge capture and ridge de-anchoring metrics. 100 Myr timeseries of lithospheric temperatures, upper mantle velocity in the vicinity of the mantle plume, and convergence velocity at nearby trenches supplementing Figure 3. Shaded regions indicate the time window of plume-ridge capture (a–c) and plume-ridge de-anchoring (d–f) from the numerical model.

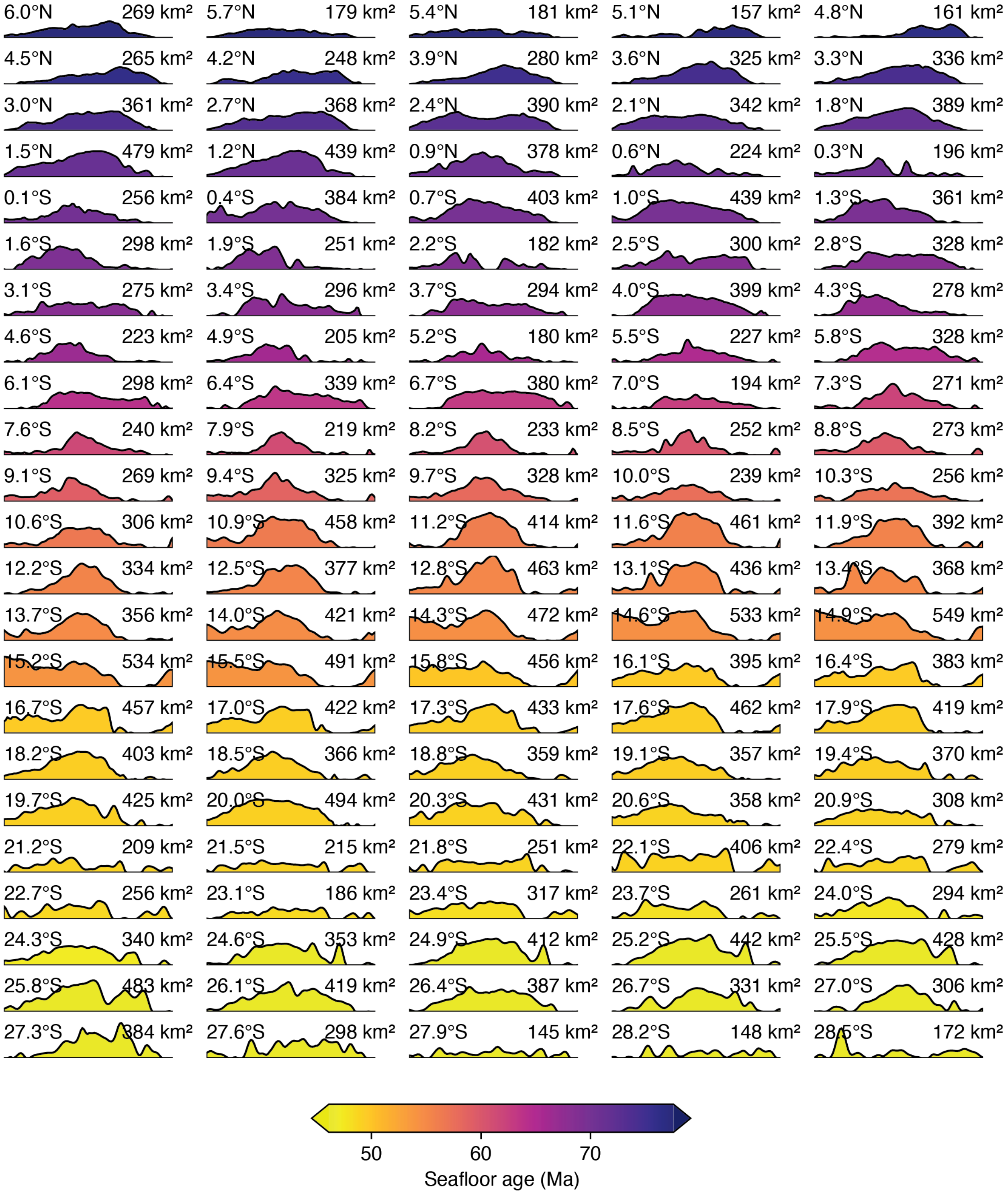

Supplementary Fig. 3: Magma flux calculated at multiple cross sections along the Ninety-East Ridge. The magma flux is calculated by subtracting the height of the ridge from the basal seafloor height (away from plume-ridge influence) and integrating the area for multiple latitudinal cross sections along the Ninety-East Ridge (see methods). Each cross section is coloured by the seafloor age and annotated by latitude and the calculated magma flux. The height of each axis is 4,280 m measured from the basal seafloor height.

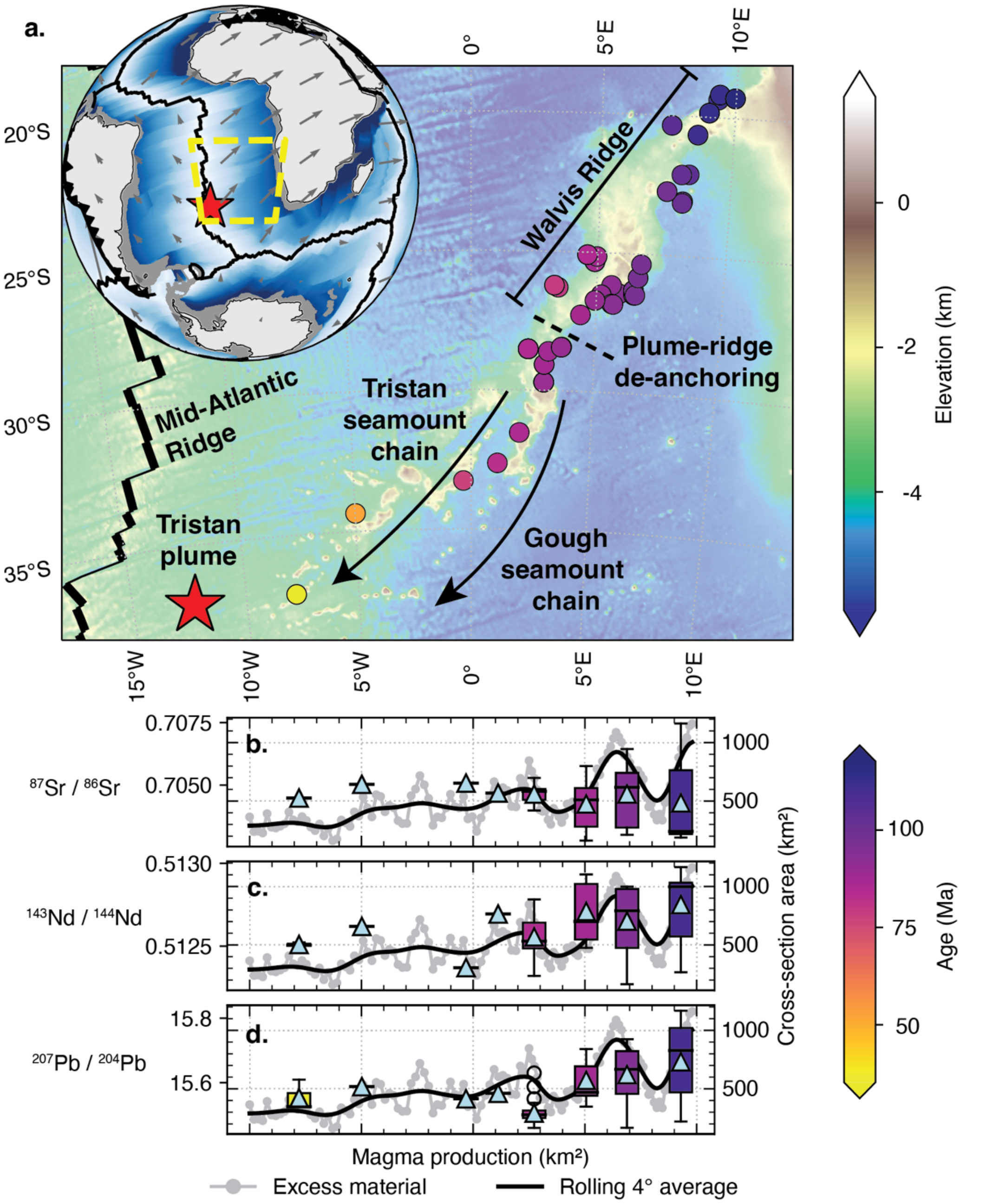

Supplementary Fig. 4: Magma flux and radiogenic enrichment along the Walvis Ridge and Tristan-Gough seamount chains. The Tristan plume was coupled to the mid-Atlantic Ridge since at least 114 Ma, producing the Walvis Ridge until plume-ridge de-anchoring at around 70 Ma[1]. Following de-anchoring, the Tristan plume produced the Tristan and Gough seamount chains from 70 Ma to the present. **a.** Map of seafloor elevation from ETOPO1[2] showing the position of the Tristan plume, the geometry of the mid-Atlantic Ridge, and age progression of the Walvis Ridge and Tristan-Gough seamount chains. **b-d.** Magma production calculated at multiple longitudinal cross-sections along the Walvis Ridge and seamount chains, overlain with isotope ratios of **b.** $^{87}Sr/^{86}Sr$, **c.** $^{143}Nd/^{144}Nd$, and **d.** $^{207}Pb/^{204}Pb$. Box plots are coloured by age, black lines indicate the median, blue triangles indicate the mean, and white circles indicate outliers. This figure replicates the workflow presented in Figure 7.

Supplementary References

1. Hoernle, K. *et al.* How and when plume zonation appeared during the 132 Myr evolution of the Tristan Hotspot. *Nature Communications* **6**, 7799 (2015).
2. Amante, C. & Eakins, B. W. ETOPO1 1 arc-minute global relief model : procedures, data sources and analysis. *NOAA Technical Memorandum NESDIS NGDC-24* https://ngdc.noaa.gov/mgg/global/global.html (2009) doi:10.7289/V5C8276M.
