# Supplementary material for: Spreading ridge migration enabled by plume-ridge de-anchoring
Source: Nat Commun. 2024 Oct 16;15:8934. doi: 10.1038/s41467-024-53397-w (PMC11484986; doi:10.1038/s41467-024-53397-w)
Supplement: Supplementary file 3 — Description of Additional Supplementary Files [file 41467_2024_53397_MOESM3_ESM.pdf]

Description of Additional Supplementary Files

Supplementary Movie 1: Numerical simulation of plume-ridge capture.

Supplementary Movie 2: Numerical simulation of plume-ridge de-anchoring
